# The association of plasma fibrinogen with clinicopathological features and prognosis in esophageal cancer patients

**DOI:** 10.18632/oncotarget.21746

**Published:** 2017-10-10

**Authors:** Fang-Teng Liu, Hui Gao, Chang-Wen Wu, Zheng-Ming Zhu

**Affiliations:** ^1^ Department of General Surgery, The Second Affiliated Hospital of Nanchang University, Nanchang 330000, Jiangxi Province, P.R. China; ^2^ Department of Urology, The Second Affiliated Hospital of Nanchang University, Nanchang 330000, Jiangxi Province, P.R. China

**Keywords:** plasma fibrinogen, esophageal cancer, prognosis, clinicopathological feature, biomarker

## Abstract

**Background:**

Numerous studies have shown that plasma fibrinogen was linked to esophageal cancer (EC) risk. However, the clinical significance of plasma fibrinogen in EC patients remain unclear and need to be further clarified.

**Results:**

A total of 2865 patients with EC from 11 published studies were included in this meta-analysis. The prognostic and clinical relevance of plasma fibrinogen were evaluated in EC patients. Statistical significance of the pooled hazard ratio (HR) was found for overall survival (OS), disease free survival (DFS) and recurrence-free survival (RFS) in EC. Subgroup analyses for OS were also performed to confirm the prognostic value of plasma fibrinogen. Additionally, the overall results indicated that elevated plasma fibrinogen was significantly associated with tumor invasion, lymph node metastasis (LNM) and clinical stage.

**Materials and Methods:**

A comprehensive literature retrieval was performed in PubMed, Embase, Cochrane database, Web of science and Chinese National Knowledge Infrastructure (CNKI) and Wanfang databases to identify relevant studies published prior to April 15, 2017.

**Conclusions:**

Elevated plasma fibrinogen could be served as a promising biomarker for predicting a poor prognosis and unfavorable clinicopathologic features for EC.

## INTRODUCTION

Esophageal cancer (EC), as one of the most aggressive cancers, has been the fifth leading cause of cancer-related deaths in China, the eighth most common cancer worldwide [1, 2]. There were two main subtypes, esophageal squamous-cell carcinoma (ESCC) and esophageal adenocarcinoma (EAC) [3]. Although the diagnosis and therapeutic method of EC have made much progress recently, most cases have been diagnosed initially at the advanced or metastatic disease stage, while the prognosis remained poor, especially for 5-year survival rate [4, 5]. Therefore, prognostic molecular markers for EC are urgently necessary with great clinical significance.

Fibrinogen, as a pro-inflammatory protein commonly involved in the process of hemostasis, has played an important role in both inflammatory responses and tumor progression and metastasis [6–8]. Numerous studies have reported that fibrinogen level in plasma was upregulated in several types of cancers and related to cancer progression and prognosis [9–14]. The roles of plasma fibrinogen on survival outcomes of EC also have been recognized, however, there were some conflicting findings. For example, Wakatsuki [15] found that plasma fibrinogen was associated with not only advanced clinicopathological factors but also the overall survival and relapse-free survival in EC patients. Wang and Takeuchi [16, 17] demonstrated that plasma fibrinogen was a valuable predictor for disease progression and prognosis in ESCC. However, Li *et al*. [18] reported that there was no significant association between preoperative plasma fibrinogen level and prognosis of ESCC. Therefore, the prognosis value of plasma fibrinogen in EC was still unclear without methodical analysis.

The aim of this work was to provide a synthetic analysis and systematic review for the role of fibrinogen in EC, and to clarify the prognostic significance and clinical relevance of plasma fibrinogen in EC patients.

## RESULTS

### Study characteristics

**Figure 1:**
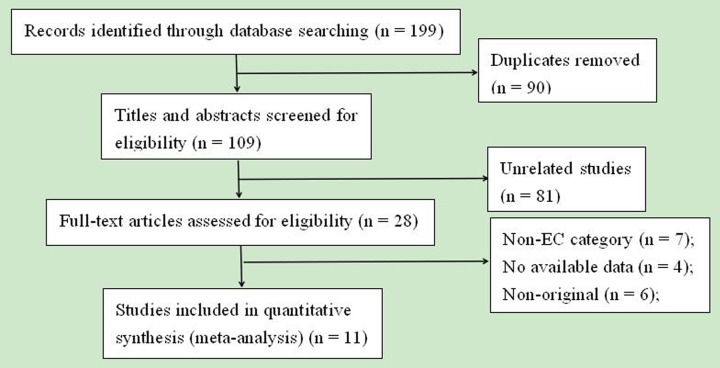
The flow diagram of the included studies

According to the inclusion and exclusion criteria mentioned above, finally, a total of 11 studies [15–25] were considered eligible for this present meta-analysis. All studies reported prognostic value of plasma fibrinogen in EC patients. The detailed selection steps were presented (Figure 1).

There were totally 2865 EC cases involved in the meta-analysis, the mean sample size was 260.5 with a minimum sample size of 68 and a maximum number of 1512. Among those included studies, 4 came from P.R. China, 6 from Japan, 1 from Austria. There were 8 studies for OS, 4 studies for DFS, 2 studies for RFS, 1 study for CSS, 1 study for LRFS and 1 study for DMFS. All recruited patients had been pathologically or histologically confirmed as EC.

Plasma fibrinogen was determined in 4 studies with a functional method based on the Clauss assay, 4 studies with automatic coagulation analyzer, 1 studies with immunoassay enzyme-linked immunosorbent assay kit, in the rest of the 2 studies, the assay was not provided. Differences in the cut-off value for determining high plasma fibrinogen were observed among the included studies. The main information of the 11 studies included in our meta-analysis was summarized (Table 1).

**Table 1:** Main characteristics of all included studies

Study (Year)	Country	No. of patients	Pathology subtype	Clinical stage	Treatments	Included period	Age median (range) (year)	Gender (M/F)	Cut-off (mg/dL)	Follow-up	Prognosis
Wakatsuki K, 2017 [15]	Japan	100	ESCC	I-IV	Surgery+ adjuvant chemotherapy (Only for patients with LNM)	1995-2006	50: ≥62 years; 50:<62 years	79/21	400	above 100 months	OS, RFS
Kijima T, 2017 [19]	Japan	98	ESCC	III/IV	Radio/Chemotherapy	2011-2014	64.9 (46–86)	86/12	400	median: 15.4 months	OS
Suzuki T, 2017 [22]	Japan	82	ESCC	I-IV	Surgery or ESD	2009-2014	67(34–87)	66/16	321	median: 28.5 months	CSS
Zhang SS, 2016 [20]	China	1512	ESCC+EA+ Others	I-IV	Surgery or Radio/Chemotherapy	2000-2008	730: >58 years; 782: ≤58 years	1144/368	400	(1-140) months	OS, DFS
Arigami T, 2015 [21]	Japan	238	ESCC	I-III	Surgery	1998-2012	65 (37–87)	210/28	400	median: 26 months	OS
Ilhan-Mutlu A, 2015 [23]	Austria	84	ESCC+EA	I-IV	Neo-adjuvant therapy+Surgery	1996-2011	mean (63 ± 9)	70/14	439.5	< 5 years	DFS
Wang J, 2015 [17]	China	119	ESCC	I-III	Surgery+ Adjuvant treatment (only 48 with Radio/Chemotherapy)	2008	60 (42-78)	95/24	400	above 60 months	OS, DFS
Zhang D, 2015 [24]	China	255	ESCC	I-IV	Surgery+ Radio/Chemotherapy	2006-2009	57 (36–81)	232/23	400	above 60 months	OS, RFS, LRFS, DMFS
Li XH, 2015 [18]	China	204	ESCC	I-IV	Surgery+ Radio/Chemotherapy (Only for some patients)	2007-2008	59 (36–79)	145/59	400	NA	OS
Matsuda S, 2014 [25]	Japan	68	ESCC	I-III	Radio/Chemotherapy/ Neo-adjuvant therapy+Surgery	2001-2010	(mean ± SD) (61.8 ± 8.11)	61/7	350	(1-100) months	DFS
Takeuchi H, 2007 [16]	Japan	105	ESCC	I-IV	Surgery/EMR+ Radio/Chemotherapy (Only for some patients)	1995-2005	68 (45–88)	90/15	350	median: 37 months	OS

ESCC: esophageal squamous cell carcinoma; EA: esophageal adenocarcinoma; OS: overall survival; DFS: disease-free survival; RFS: relapse-free survival; CSS: cause-specific survival; LRFS: locoregional relapse-free survival; DMFS: distant metastasis free survival; LNM: lymph node metastasis; ESD:endoscopic submucosal dissection; EMR:endoscopic mucosal resection.

### Relationship between plasma fibrinogen and EC prognosis/OS in EC

**Figure 2:**
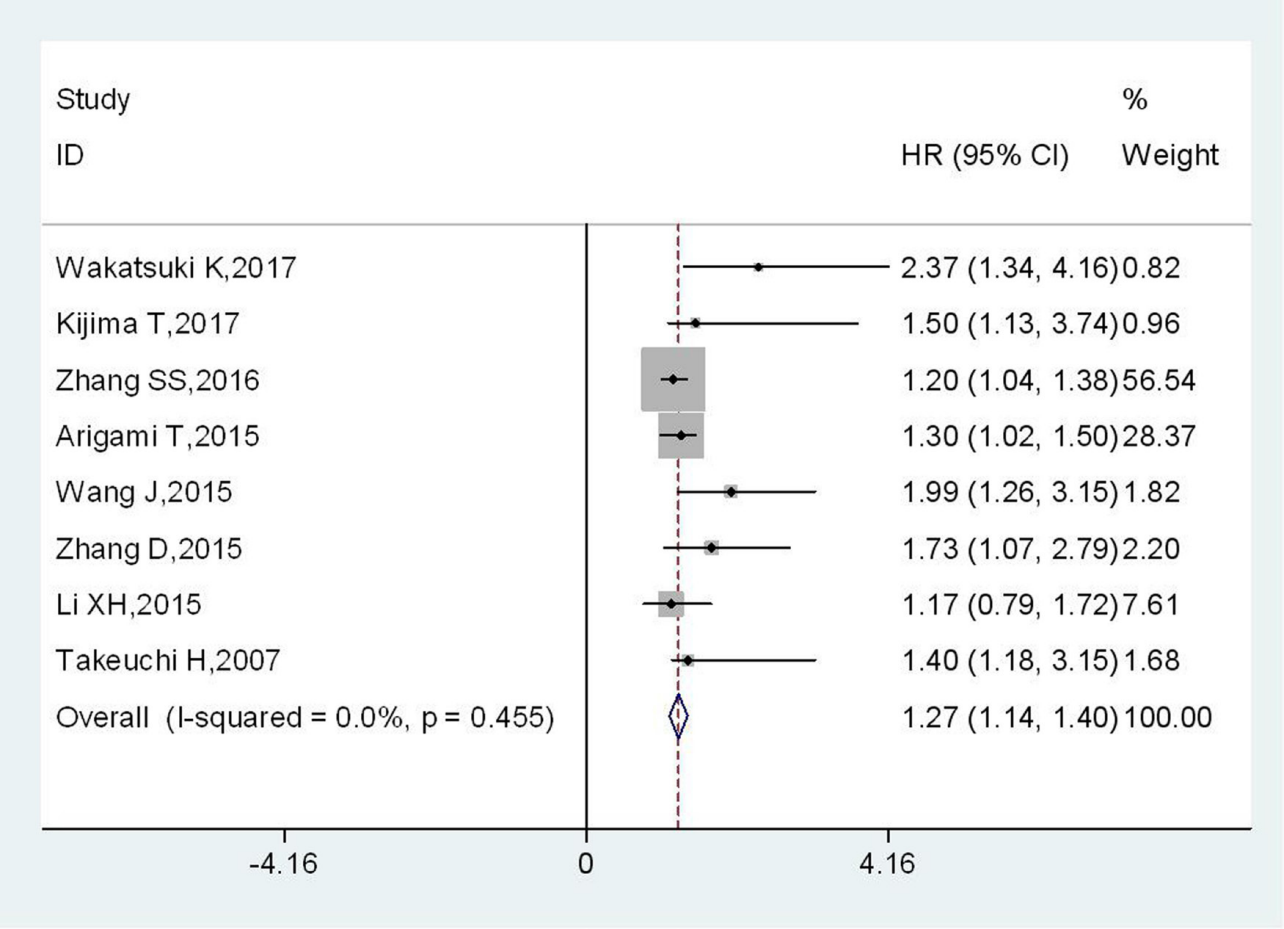
Forest plot of HR for the relationship between plasma fibrinogen and OS in EC

A total of 8 studies, including 2631 EC cases, reported the OS corresponding to the level of plasma fibrinogen. There was no significant heterogeneity among studies (I^2^=0.0%, P_h_=0.455), thus the fixed-effects model was adopted to evaluate the pooled HRs with corresponding 95% CIs. The overall results demonstrated that EC patients with increased plasma fibrinogen showed a significantly poor OS compared to those with lower plasma fibrinogen (HR:1.27, 95% CI:1.14-1.40, *p*<0.001) (Figure 2).

Further, we performed subgroup analyses for OS by the pathology subtype, country, cut-off value, sample size and analysis type. Statistically significant pooled HR values >1 were also consistently calculated in those subgroup meta-analyses (Table 2, those figures are presented in the Supplementary Materials).

**Table 2:** Summary of the meta-analysis results of pooled HRs of OS of EC patients with elevated plasma fibrinogen

Analysis	No. of studies	No. of patients	Pooled HR	*p*-value	Heterogeneity	
			(95% CI)		I^2^ (%)	P_h_
[1] OS	8	2631	1.27 (1.14–1.40)	<0.001	0.0	0.455
[2] Pathology subtype						
ESCC	7	1119	1.36 (1.16–1.55)	<0.001	0.0	0.499
Mixed	1	1512	1.20 (1.04–1.38)	0.012	-	-
[3] Country						
China	4	2090	1.23 (1.08–1.39)	<0.001	24.3	0.265
Japan	4	541	1.34 (1.11-1.57)	<0.001	0.0	0.526
[4] Cut-off value						
400 mg/dL	7	2526	1.27 (1.14-1.39)	<0.001	10.2	0.351
350 mg/dL	1	105	1.40 (1.18-3.15)	0.006	-	-
[5] Sample size						
≥ 200	4	2209	1.24 (1.11-1.37)	<0.001	0.0	0.618
< 200	4	422	1.77 (1.22-2.33)	<0.001	0.0	0.656
[6] Analysis type						
Multivariate	3	1867	1.24 (1.07-1.40)	<0.001	48.8	0.142
Non-multivariate	5	764	1.31 (1.11-1.52)	<0.001	0.0	0.647

### Plasma fibrinogen and DFS in EC

**Figure 3:**
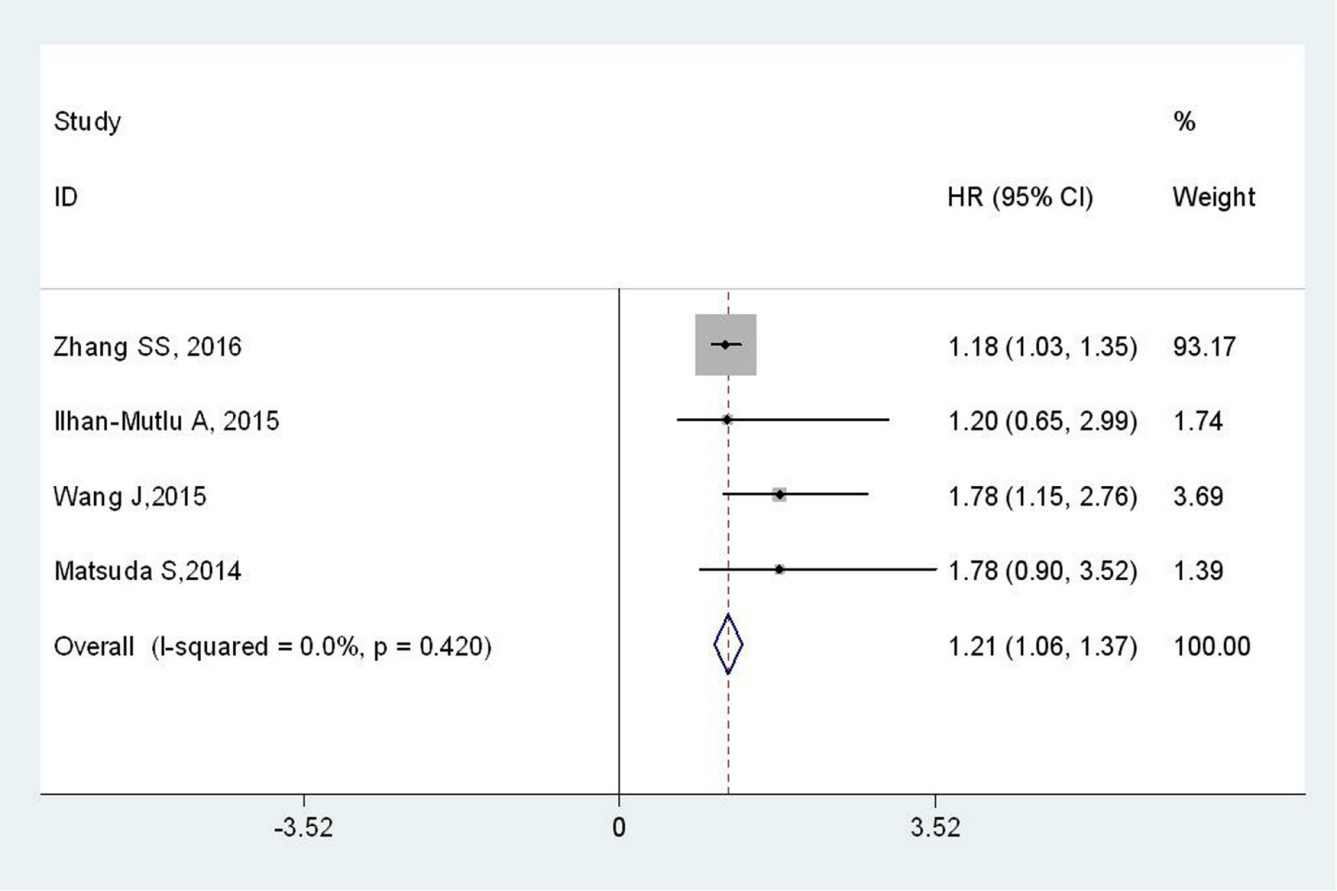
Forest plot of HR for the relationship between plasma fibrinogen and DFS

A total of 4 studies, comprising 1783 EC patients, explored the relationship between plasma fibrinogen and DFS. No significant heterogeneity was observed among studies (I^2^ = 0.0 %; P_h_ = 0.420), the fixed-effects model was applied. The pooled results showed DFS was significantly worse in EC patients with high plasma fibrinogen (HR = 1.21; 95% CI = 1.06–1.37; *p* < 0.001) (Figure 3).

### Plasma fibrinogen and RFS in EC

**Figure 4:**
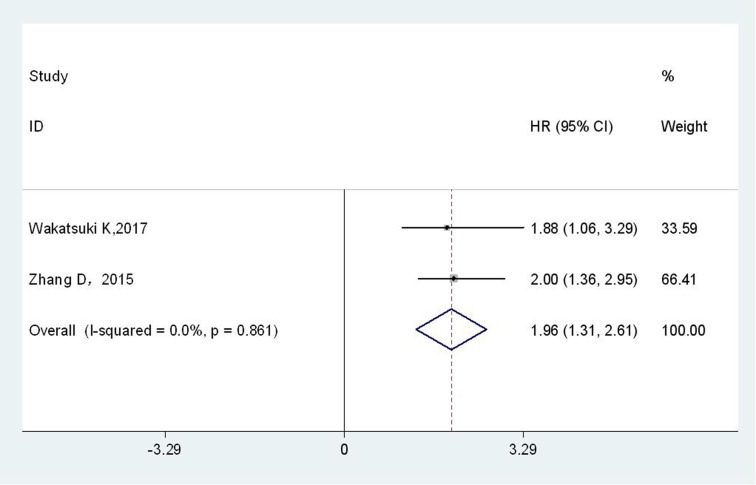
Forest plot of HR for the relationship between plasma fibrinogen and RFS

Only two studies, with a total of 355 patients, provided available information for RFS analysis. The fixed-effects model was applied since there was no significant heterogeneity across-studies (I^2^ = 0.0 %; P_h_ = 0.861). As shown in Figure 4, EC patients with higher plasma fibrinogen level had a worse RFS compared with those with lower plasma fibrinogen (HR = 1.96; 95% CI = 1.31-2.61; *p* < 0.001).

### Association between plasma fibrinogen and clinical parameters in EC

Regarding the clinical significance of plasma fibrinogen in EC patients, the pooled results indicated that elevated plasma fibrinogen was significantly associated with the depth of tumor invasion (OR = 3.26, 95% CI: 1.75-6.07), lymph node metastasis (OR = 1.79, 95% CI: 1.48-2.16) and clinical stages (OR = 3.23, 95% CI: 2.02-5.16). But no significant correlation was observed between plasma fibrinogen with gender or tumor differentiation. The detailed results for plasma fibrinogen and clinicopathological parameters were provided (Table 3, those figures are presented in the Supplementary Materials).

**Table 3:** Meta-analysis of the association between elevated plasma fibrinogen and clinicopathologic features in EC patients

Clinicopathologic features	Studies(n)	No. of patients	OR (95% CI)	*p*-value	Heterogeneity		
					I^2^ (%)	P_h_	Model
Gender (Male vs. Female)	6	2136	1.53(0.83-2.82)	0.17	55	0.05	Random
Tumor invasion (T3-T4 vs.T1-T2)	6	2136	3.26(1.75-6.07)	0.0002	78	0.0004	Random
Tumor differentiation (G2-G3 vs. G1)	4	1986	1.00(0.81-1.24)	0.99	0	0.77	Fixed
Lymph node metastasis (Yes vs. No)	6	2136	1.79(1.48-2.16)	<0.00001	14	0.33	Fixed
TNM stage (III-IV vs. I-II)	4	369	3.23(2.02-5.16)	<0.00001	0	0.46	Fixed

### Publication bias

**Figure 5:**
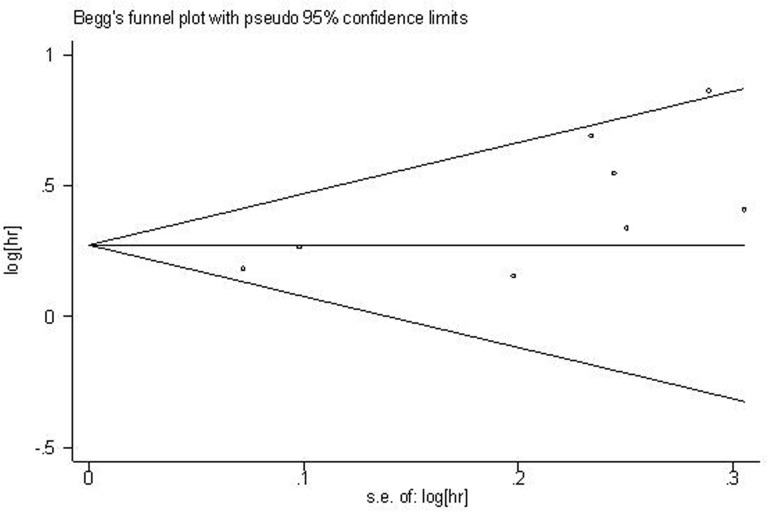
Funnel plot analysis of potential publication bias for OS

**Figure 6:**
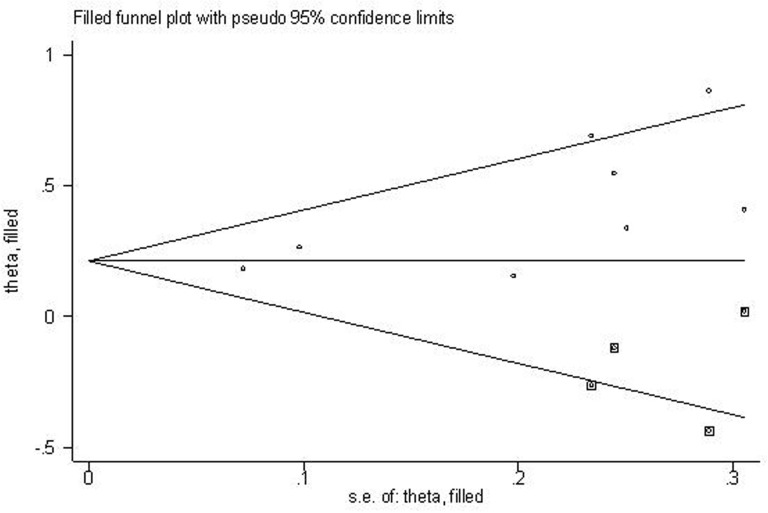
Filled funnel plot of meta-analysis using “trim-and-fill” method “○” indicates observed studies; “◘” indicates missed studies.

For the meta-analysis of the association between plasma fibrinogen and OS, Begg's funnel plot was provided (Figure 5). The result from Begg's test showed that there was no significant publication bias across-studies (for Begg's test: z= 1.61 (continuity corrected); Pr > |z| = 0.108 (continuity corrected)). However, the result from Egger's test showed a potential publication bias existed (P>|t|=0.028, 95%CI:0.258-3.178), then the “trim and fill method” was applied to replace four missing studies (Figure 6). After correction, the adjusted pooled HR was 1.236 (95 % CI: 1.127- 1.355, *p* < 0.001).

### Sensitivity analysis

**Figure 7:**
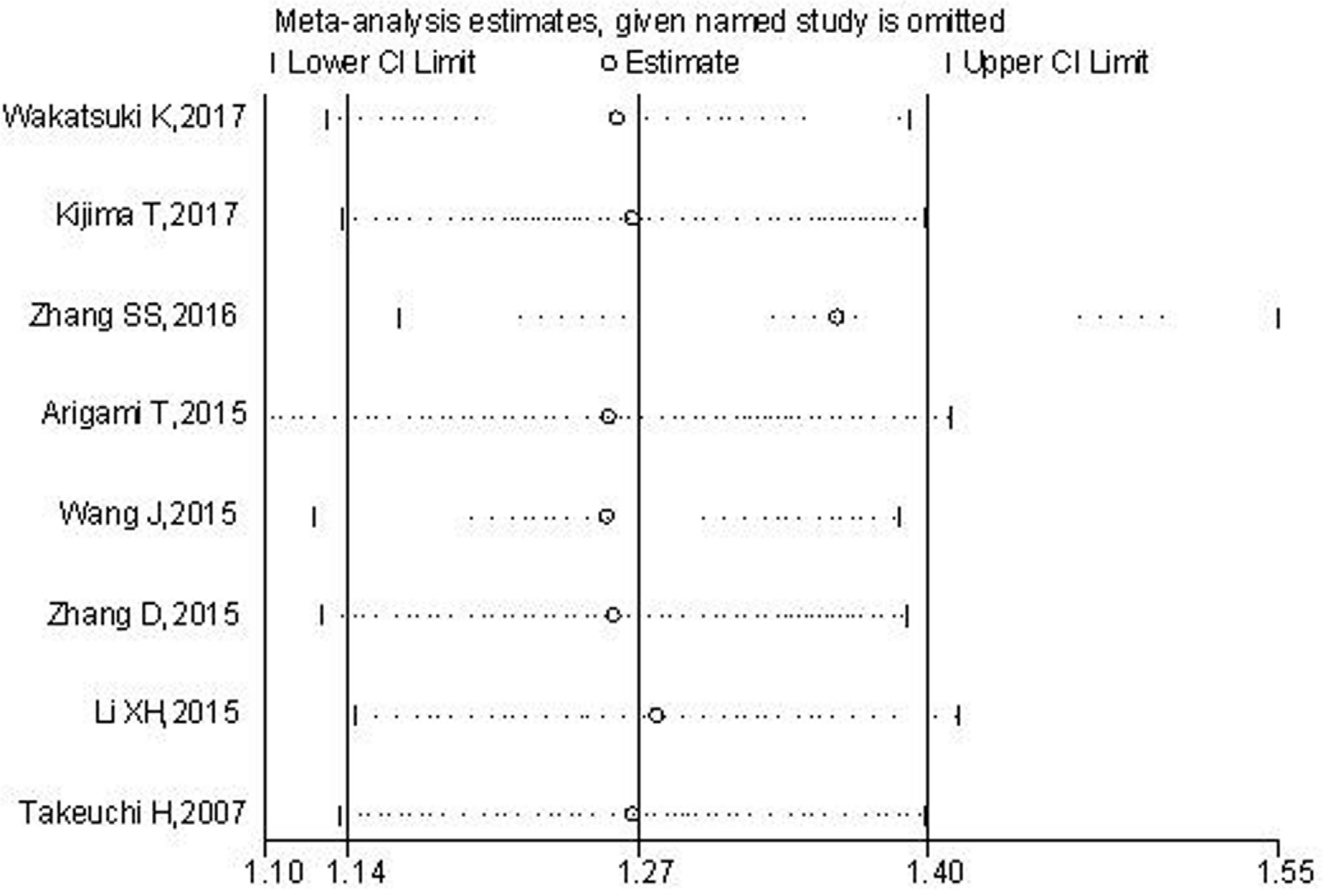
Sensitivity analysis of the pooled HRs of plasma fibrinogen and OS in EC

Sensitivity analysis was conducted to assess the effect of each single study on OS. It showed that the combined result was not significantly altered after the exclusion of any studies (Figure 7).

## DISCUSSION

A number of studies have reported various results relating hyperfibrinogenemia to EC risk of prognosis and clinical relevance. However, up to now, few meta-analysis has been performed to synthetically assess the clinical and prognostic value of plasma fibrinogen in EC patients.

In our current study, a total of 11 studies was combined to validate prognostic value of plasma fibrinogen in EC. The synthesis analysis showed that hyperfibrinogenemia was associated with clinical progression and worse survival in EC patients. The patients with higher plasma fibrinogen showed a shorter OS, poorer DFS and worse RFS when compared with those with lower expression level. Subgroup analyses for OS were also conducted to confirm the prognostic significance of plasma fibrinogen in EC. Furthermore, we explored the correlation between plasma fibrinogen level and some clinicopathological characteristics. EC patients with elevated plasma fibrinogen level were more likely to be with deeper tumor invasion, positive lymph node metastasis and advanced tumor stages. But there was no significant correlation between plasma fibrinogen with gender or tumor differentiation. Taken together, elevated plasma fibrinogen was associated with tumor progression and poor survival in patients with EC, and it may serve as a valuable predictive factor for EC clinicopathology and prognosis.

The value in prognostic evaluation of plasma fibrinogen could be interpreted by its biological mechanism in EC patients from the important association between hemostatic factors and cancer progression. Inflammatory response has been believed to participate in the tumor progression, it could cause the elevated levels of various inflammatory cytokines [26–28]. Fibrinogen was a P-globulin, belong to pro-inflammatory protein. Fibrinogen participated in the formation of extracellular matrix and could be endogenously synthesized by cancer cells in addition to liver cells [29–31]. Fibrinogen played a key role in the development of tumor. It could regulate the growth of cancer cells by binding to the growth factors, such as vascular endothelial growth factor (VEGF) and platelet-derived growth factor (PDGF) [30–32]. Moreover, elevated fibrinogen could enhance the cell migration and invasion by induced epithelial-mesenchymal transition (EMT) via regulating the expression level of vimentin and Ecadherin [33]. It was also demonstrated that fibrinogen played an important role in the oncogenesis and development of tumor by aggravating cell proliferation, inhibiting apoptosis and stimulating angiogenesis and hematogenous metastasis [30, 31, 34–36]. The fibrinogen would also be affected by the chemotherapy and radiotherapy, through the trigger of inflammatory response and immune response.

Our meta-analysis was the first study to systematically investigate the clinical and prognostic value of plasma fibrinogen level in EC. The elevated level of plasma fibrinogen could predict poorer pathological features and was a significant risk factor affecting survival in EC patients.

However, several limitations of this study need to be acknowledged. Firstly, the number of included studies was relatively small and total sample size should be enlarged. Secondly, the cut-off value applied was varied in studies and the detection methods for plasma fibrinogen were not identical. Thirdly, most of patients enrolled came from Asia countries, and only one study included was from western country, this might limit the applicability of our findings for other ethnic groups. Fourthly, publication bias may exist, despite no significant publication bias was observed based on the trim and fill method, as well as stable results shown in sensitivity analysis. Finally, other factors might also play roles in EC prognosis, such as clinical stage and treatment.

In conclusion, our study provided a strong evidence that elevated plasma fibrinogen was closely associated with unfavorable prognosis and aggressive clinical features in patients with EC. Certainly, well-designed clinical researches on larger sample and other ethnic groups are needed to further validate of our study.

## MATERIALS AND METHODS

### Search strategy

We performed a comprehensive literature retrieval for published original articles in a number of online database: PubMed, Embase, Cochrane databases, Web of science and Chinese National Knowledge Infrastructure (CNKI) and Wanfang database. The retrieval was updated until April 15, 2017.

Retrieval was performed with following key search items: fibrinogen (e.g., “plasma fibrinogen”), esophageal cancer (e.g., “oesophageal cancer”, “esophageal carcinoma”, “EC”, “esophageal squamous cell carcinoma”, “ESCC”, “esophageal adenocarcinoma”) and prognosis (e.g., “prognostic”, “survival”, “outcome”, “recurrence”). In addition, references of relevant publications were manually reviewed for potential eligible studies.

### Selection criteria and definitions

The eligible articles were included only if they met the following criteria: (1) Clinical study investigated the prognostic effect of plasma fibrinogen on esophageal cancer; (2) the patients were divided into two groups according the level of plasma fibrinogen; (3) the hazard ratios with corresponding 95 % CIs for prognosis were provided or could be manually calculated; (4) articles were published in English or Chinese.

Articles with the following criteria were excluded: (1) Reviews and non-original articles; (2) studies referred other kinds of human cancers; (3) studies lacking sufficient data to collect HRs with 95% CIs.

### Data extraction and study quality

The data and information were extracted from those included studies independently by two investigators (GH and LFT), including: the name of first author, publication year, country, sample size, pathology subtype, clinical stage, recruitment period, age of patients, gender ratio, cut-off value, follow-up time, hazard ratio (HR) and corresponding 95 % confidence interval (CI) for prognosis and relevant clinicopathological data.

If a study provided the results of both multivariate outcome and univariate outcome, we chose the former. If a study only provided Kaplan-Meier survival curves, then we extracted survival data from that via Engauge Digitizer V4.1. The relevant clinicopathologic characteristics were extracted directly from the original articles.

For quality assessment, the Newcastle-Ottawa Scale (NOS) was applied to assess the quality of studies. The NOS score was ranged from 0 to 9. Studies with NOS score ≥ 6 was graded as high quality. The quality of all including studies in this meta-analysis was varied from 6 to 9, with a mean value of 6.5.

### Statistical analysis

The pooled HR and its corresponding 95% CI for prognosis were calculated with Stata SE12.0. The combined ORs and its corresponding 95% CI for clinical parameters were calculated by RevMan5.3 software.

Statistical heterogeneity among studies was assessed with I^2^test and Q statistic test, the fixed-effect model was applied when no obvious heterogeneity was observed among studies, otherwise, the random effects model was applied to calculate parameters when there was significant heterogeneity across studies(I^2^ > 50% or P_h_<0.05 suggested significant heterogeneity).

Funnel plots and Begg's test/Egger's test were involved to search the potential publication bias. The sensitivity analysis was also performed to test the reliability of the combined results. A *p* value less than 0.05 was considered statistically significant.

## SUPPLEMENTARY MATERIALS FIGURES


