# Supplementary material for: The association of plasma fibrinogen with clinicopathological features and prognosis in esophageal cancer patients
Source: Oncotarget. 2017 Oct 10;8(54):93029–38. doi: 10.18632/oncotarget.21746 (PMC5696241; doi:10.18632/oncotarget.21746)
Supplement: Supplementary file 1 [file oncotarget-08-93029-s001.pdf]

The association of plasma fibrinogen with clinicopathological features and prognosis in esophageal cancer patients

SUPPLEMENTARY MATERIALS

(1) Plasma fibrinogen and OS in EC

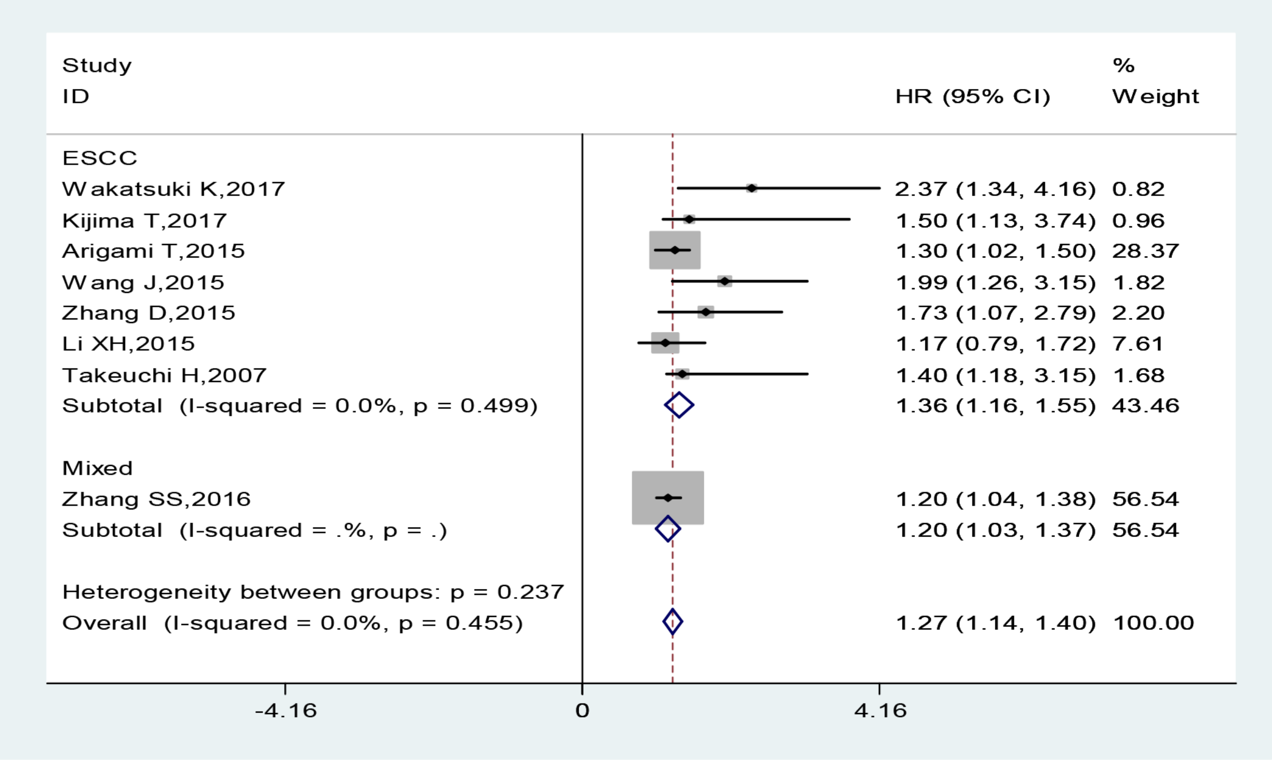

Supplementary Figure 1: Meta-analysis for the pooled HRs of OS stratified by the pathology subtype.

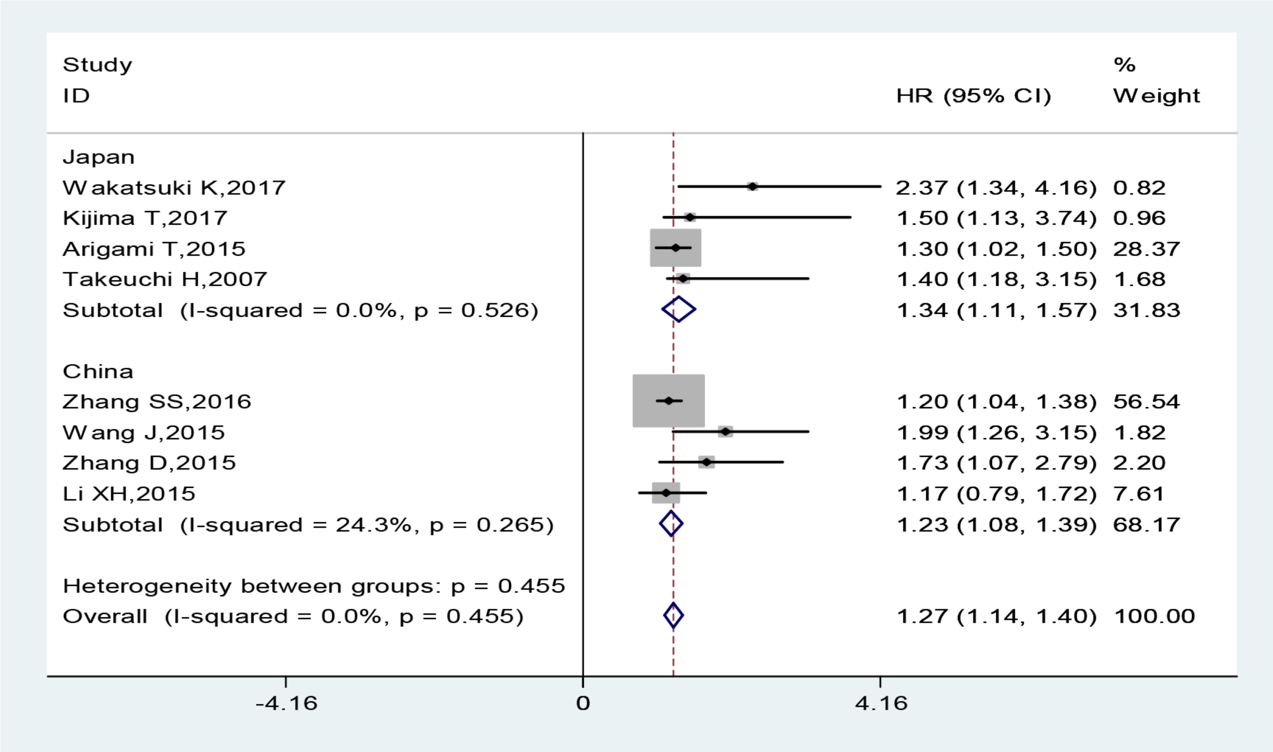

Supplementary Figure 2: Meta-analysis for the pooled HRs of OS stratified by country.

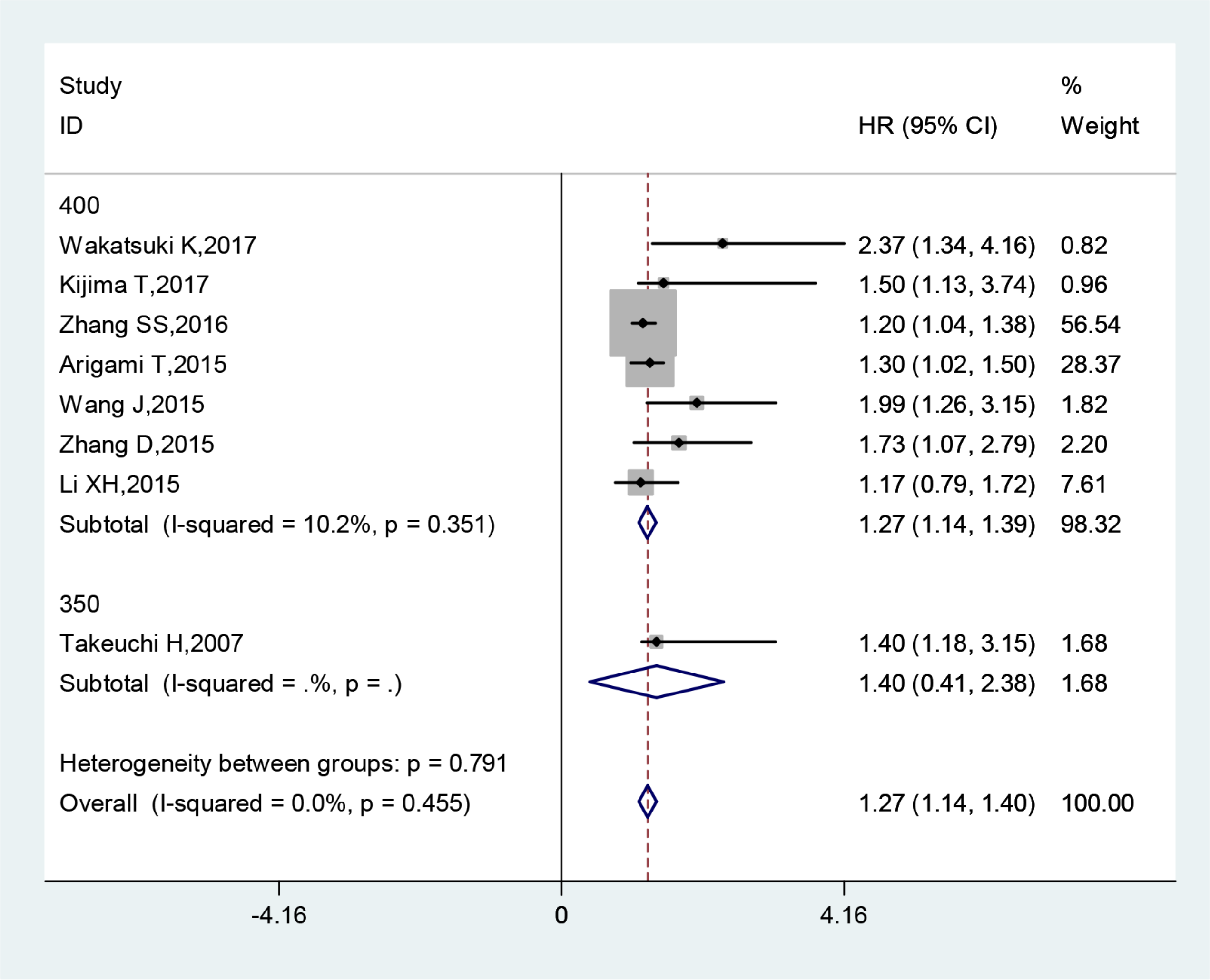

Supplementary Figure 3: Meta-analysis for the pooled HRs of OS stratified by the cut-off value.

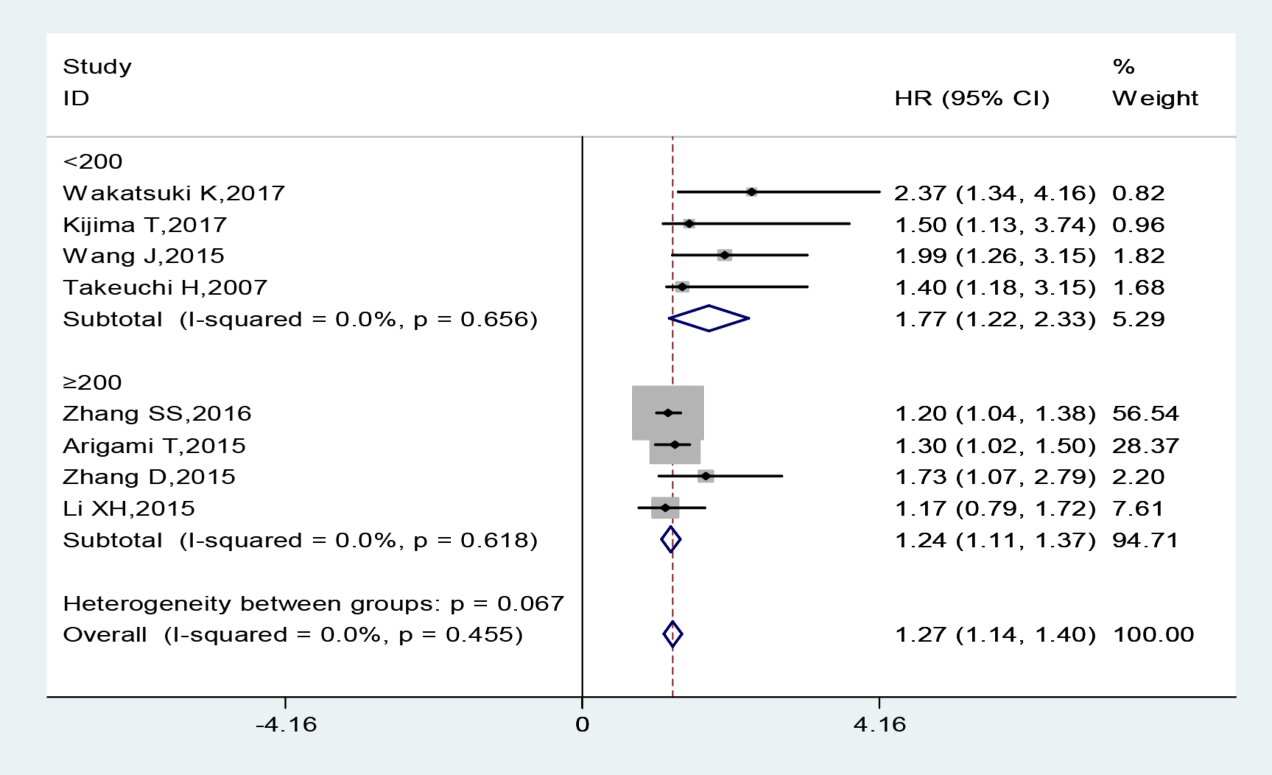

Supplementary Figure 4: Meta-analysis for the pooled HRs of OS stratified by the sample size.

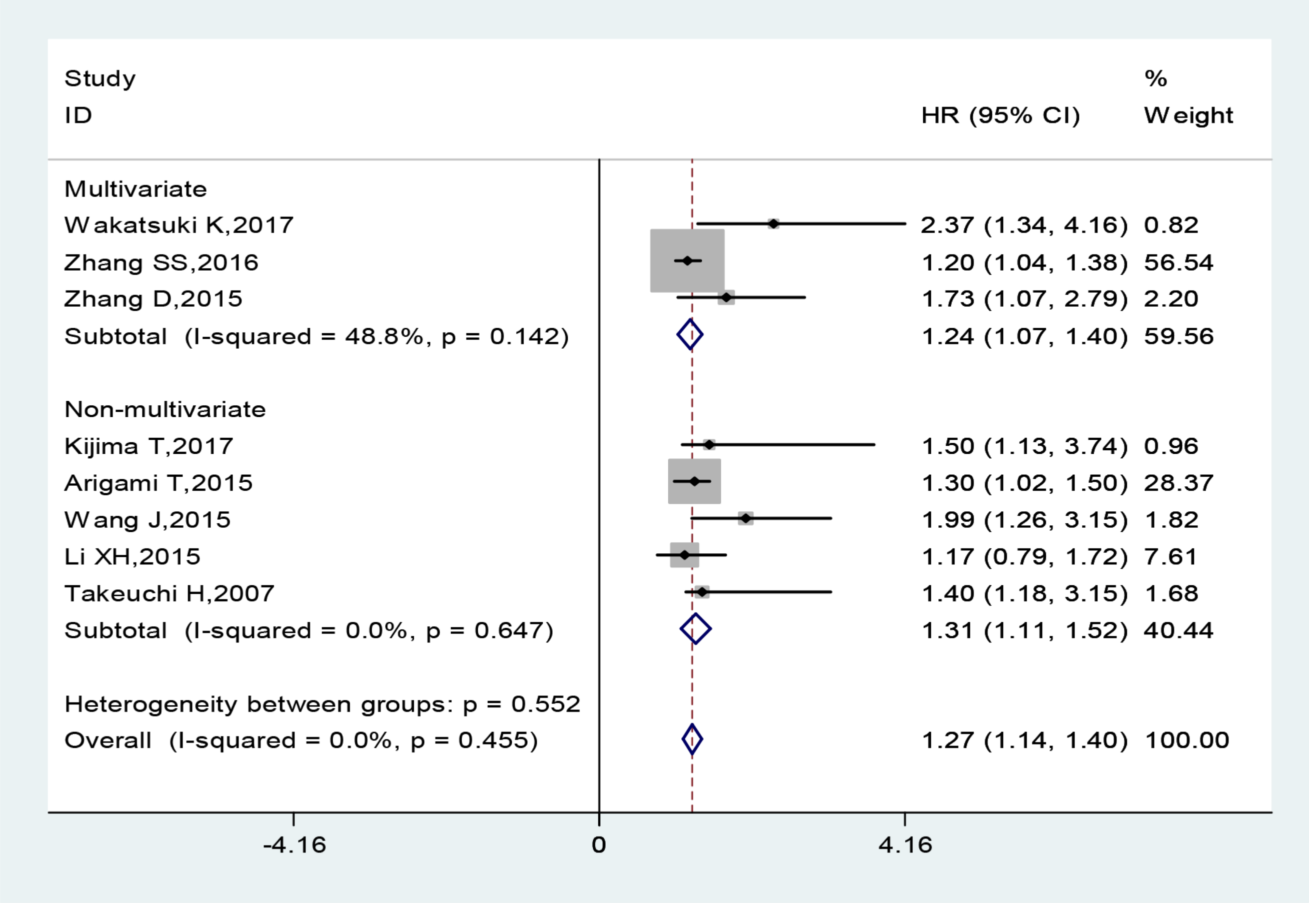

Supplementary Figure 5: Meta-analysis for the pooled HRs of OS stratified by the analysis type.

(2) Association between plasma fibrinogen and clinical parameters in EC

	Male		Female			Odds Ratio	
Study or Subgroup	Events	Total	Events	Total	Weight	M-H, Random, 95% CI	Year
Matsuda S,2014	33	61	5	7	9.3%	0.47 [0.08, 2.62]	2014
Wang J,2015	44	95	8	24	19.2%	1.73 [0.67, 4.42]	2015
Zhang D,2015	99	232	4	23	16.2%	3.54 [1.17, 10.72]	2015
Zhang SS,2016	360	1144	85	368	32.9%	1.53 [1.16, 2.01]	2016
Wakatsuki K,2017	26	79	1	21	7.0%	9.81 [1.25, 77.17]	2017
Suzuki T,2017	34	66	11	16	15.4%	0.48 [0.15, 1.54]	2017
**Total (95% CI)**		**1677**		**459**	**100.0%**	**1.53 [0.83, 2.82]**	
Total events	596		114				

Heterogeneity: $Tau^2 = 0.27$; $Chi^2 = 11.02$, $df = 5$ ($P = 0.05$); $I^2 = 55\%$

Test for overall effect: $Z = 1.37$ ($P = 0.17$)

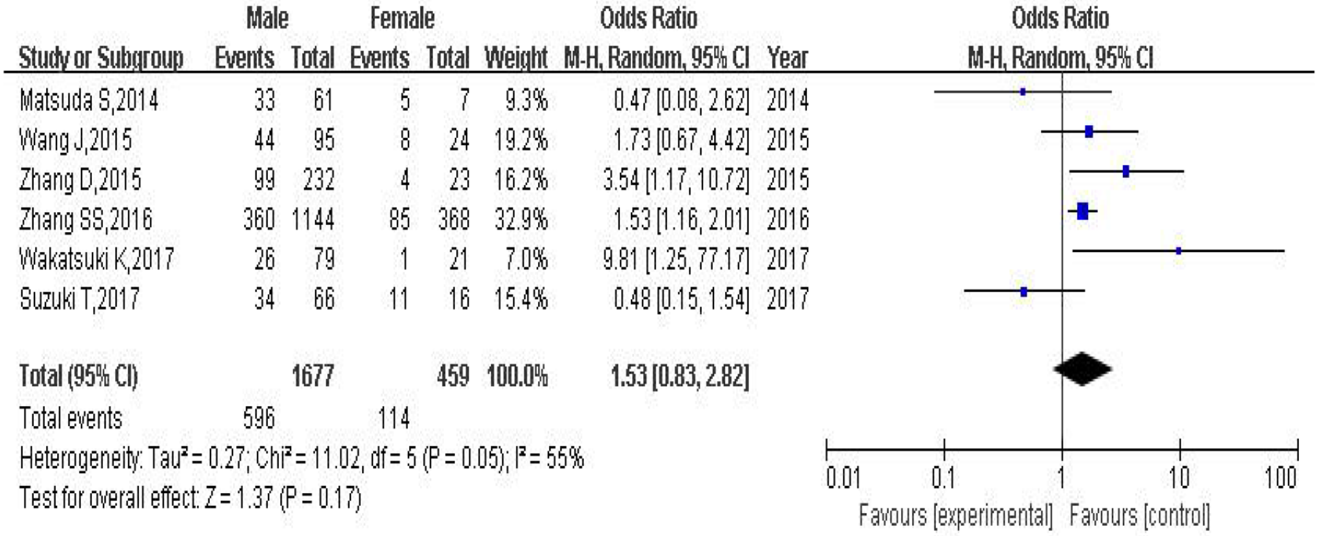

Supplementary Figure 6: The correlation between plasma fibrinogen and gender.

	T3-T4		T1-T2			Odds Ratio	
Study or Subgroup	Events	Total	Events	Total	Weight	M-H, Random, 95% CI	Year
Matsuda S,2014	27	40	11	28	14.6%	3.21 [1.17, 8.78]	2014
Zhang D,2015	89	170	14	85	18.8%	5.57 [2.92, 10.65]	2015
Wang J,2015	40	75	12	44	16.9%	3.05 [1.36, 6.81]	2015
Zhang SS,2016	333	1050	112	462	22.7%	1.45 [1.13, 1.86]	2016
Wakatsuki K,2017	21	58	6	42	14.5%	3.41 [1.23, 9.41]	2017
Suzuki T,2017	20	24	25	58	12.6%	6.60 [2.00, 21.75]	2017
**Total (95% CI)**		**1417**		**719**	**100.0%**	**3.26 [1.75, 6.07]**	
Total events	530		180				

Heterogeneity: $Tau^2 = 0.43$; $Chi^2 = 22.82$, $df = 5$ ($P = 0.0004$); $I^2 = 78\%$

Test for overall effect: $Z = 3.71$ ($P = 0.0002$)

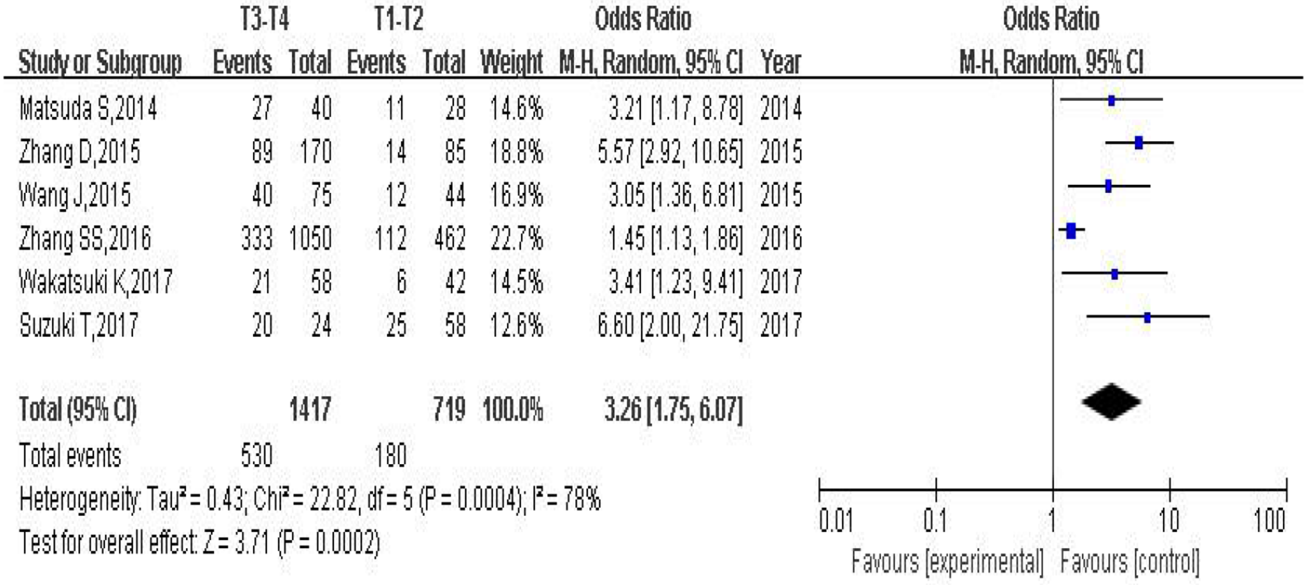

Supplementary Figure 7: The correlation between plasma fibrinogen and tumor invasion.

	G2-3		G1			Odds Ratio	
Study or Subgroup	Events	Total	Events	Total	Weight	M-H, Fixed, 95% CI	Year
Zhang D,2015	93	225	10	30	6.0%	1.41 [0.63, 3.15]	2015
Wang J,2015	36	86	16	33	7.8%	0.77 [0.34, 1.71]	2015
Zhang SS,2016	146	497	299	1015	80.5%	1.00 [0.79, 1.26]	2016
Wakatsuki K,2017	15	56	12	44	5.7%	0.98 [0.40, 2.37]	2017
**Total (95% CI)**		**864**		**1122**	**100.0%**	**1.00 [0.81, 1.24]**	
Total events	290		337				

Heterogeneity: $Chi^2 = 1.13$, df = 3 (P = 0.77); $I^2 = 0\%$

Test for overall effect: Z = 0.02 (P = 0.99)

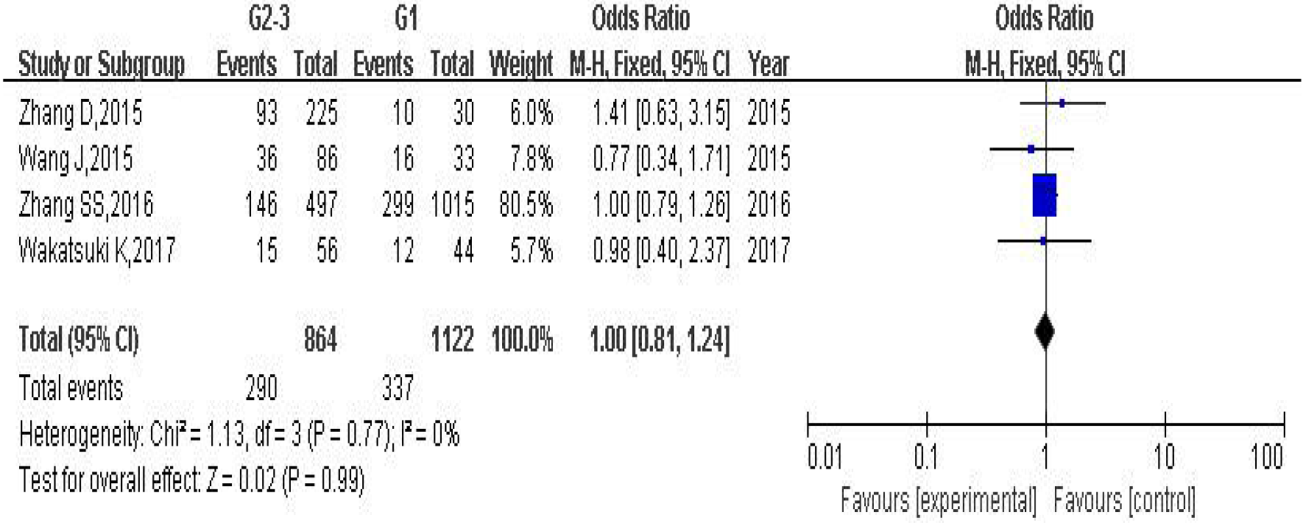

Supplementary Figure 8: The correlation between plasma fibrinogen and tumor differentiation.

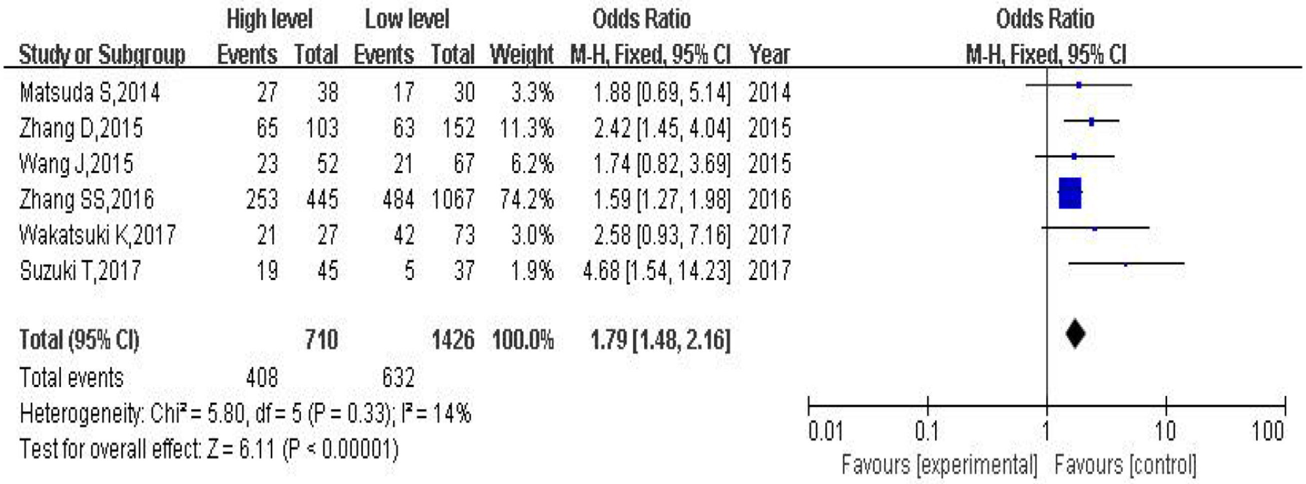

Supplementary Figure 9: The correlation between plasma fibrinogen and lymph node metastasis.

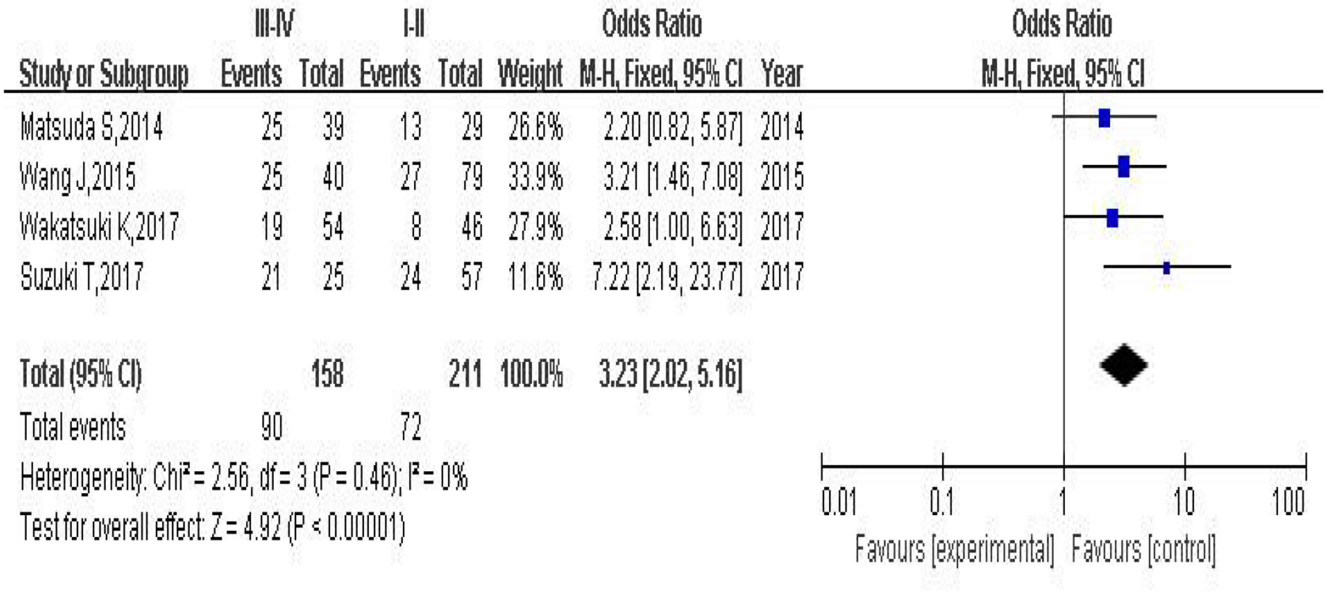

Supplementary Figure 10: The correlation between plasma fibrinogen and TNM stage.
